# Clonality Assessment in a Case of Multifocal Adamantinoma and a Review of the Literature

**DOI:** 10.1155/2012/605685

**Published:** 2012-10-09

**Authors:** Paul Borbas, Andreas Leithner, Patrick Sadoghi, Anne Berndt, Bernadette Liegl, Oskar A. Haas

**Affiliations:** ^1^Department of Orthopedic Surgery, Medical University of Graz, 8036 Graz, Austria; ^2^Children's Cancer Research Institute (CCRI), St. Anna Children's Hospital, 1090 Vienna, Austria; ^3^Institute of Pathology, Medical University of Graz, 8036 Graz, Austria

## Abstract

Adamantinoma is a low-grade, malignant biphasic bone tumour predominantly located in the tibia. In up to 50% of all cases this is combined with one or more lesions in the ipsilateral fibula. Whether these lesions represent regional metastases or arise de novo is not yet exactly known. In order to address this question, we extracted DNA from the respective fresh frozen tumour tissues in a case of a young woman with a multifocal adamantinoma of both the tibia and ipsilateral fibula. Afterwards the X inactivation pattern was studied by means of methylation-sensitive polymerase chain reaction and primers that target the polymorphic CGG trinucleotide repeat of FMR1 gene and the polymorphic CAG repeat, on exon 1 of the human androgen receptor gene (AR). The analysis of the AR was homozygous and not informative. Studying the FMR1 gene, we detected a 100% skewing of the X inactivation pattern of both locations and found that the same allele was methylated. Even if the fibula lesion arose de novo there would have been a 50 : 50 chance that the same allele was methylated. As this methylation pattern was found we cannot provide a valid explanation for the origin of the fibula lesion. Analysis of X inactivation patterns in future cases of polyfocal adamantinoma might provide further evidence for one of the two theories.

## 1. Background

According to the WHO classification of tumours, published in 2002, adamantinoma is a low-grade, malignant biphasic tumour characterized by a variety of morphological patterns, most commonly epithelial cells, surrounded by relatively bland spindle-cell osteofibrous components [1]. Adamantinoma comprises about 0.4% of all primary bone tumours with a median patient age of 25–35 years. A slight predominance of male patients has been reported [2]. At the moment there are considered to be two subtypes of adamantinomas: the so-called “classical” type and the more benign “differentiated” form [3] which is also known as “osteofibrous dysplasia-like” subtype [1].

Adamantinoma may sometimes be difficult to distinguish histologically from osteofibrous dysplasia, an important distinction which can be made by using clinical information such as patient age and history as well as by considering the location in the tibial diaphysis [2, 3]. Treatment of choice for this low-grade and radioresistant tumour is surgery whereby the use of a vascularized fibula autograft with a tibial allograft shows excellent results [4, 5] and is therefore considered to be the treatment of choice. The tibia, in particular the anterior (meta-) diaphysis, is involved in 85–90% of cases [1]. Varying in the literature from 10 to 50%, this is also combined with one or more lesions in the ipsilateral fibula. In rare cases, other sites have been reported such as ulna, humerus, femur, ribs, spine, and the short bones of the feet [1, 2, 6].

However, there are, up to now, no reports as to whether the lesion in the ipsilateral fibula represents a regional metastasis or arises de novo. We, therefore, set out to investigate this question by determining the X inactivation pattern in a case of a multifocal adamantinoma with an involvement of the ipsilateral fibula.

## 2. Case Presentation

**Figure 1:**
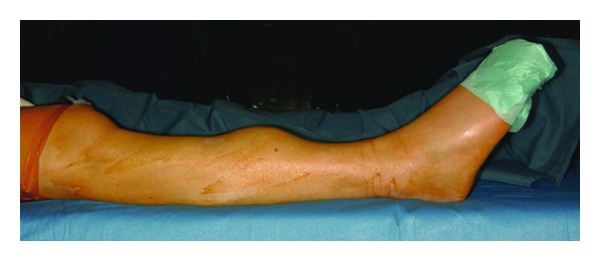
Preoperative clinical aspect of the right lower leg in a 23-year-old female with a multifocal adamantinoma of the tibia and the fibula.

**Figure 2:**
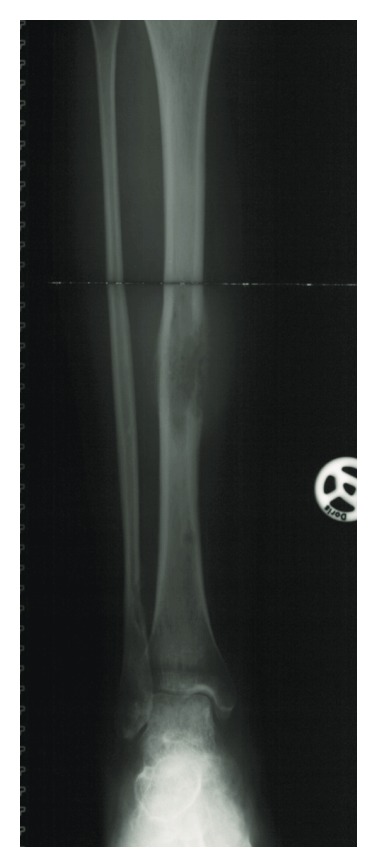
Preoperative AP radiograph of the right lower leg of a 23-year-old female patient with a multifocal adamantinoma of the tibia and the fibula.

After 7 years of symptoms a polyfocal adamantinoma was diagnosed by needle biopsy in the right lower leg of a 23-year-old female. Clinically, the patient was sensitive to pressure in the swollen middle area of the tibia as well as in the zone of the fibula (Figures 1 and 2). Magnetic resonance imaging (MRI) revealed a main lesion at the described location of the tibia which measured 7.5 × 4.0 × 3.8 cm. Furthermore, 4 cm below the caudal end of the main lesion, a tibial expansion with a maximum diameter of 1 cm as well as a 2.5 × 2.0 × 2.5 cm expansion in the medial cortex of the distal fibular metaphysis was detected on MRI.

**Figure 3:**
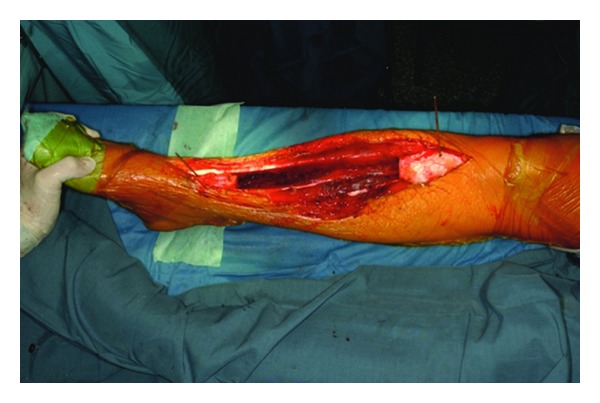
Intraoperative photograph after the wide resection of the adamantinoma (21 cm of the tibia, 19 cm of the fibula) was performed.

**Figure 4:**
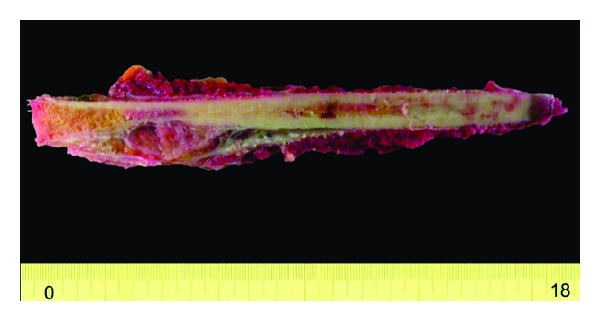
Macroscopic preparation that shows the resected 21 cm of the tibial area.

**Figure 5:**
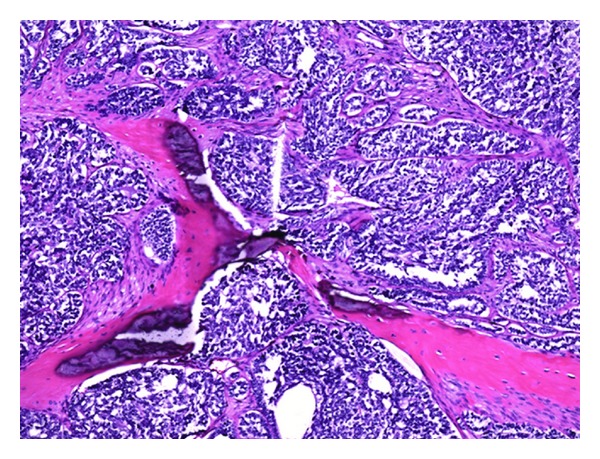
Histological preparation (100x magnification, H-E stain) showing the tumour, which infiltrates between preexisting bone-trabeculae. The tumour cells have scanty cytoplasm and a chromatin rich nucleus and are similar to basaloid cells. The tumour shows solid areas and tubular structures here. Fibrous septae cross the basaloid cell aggregates.

**Figure 6:**
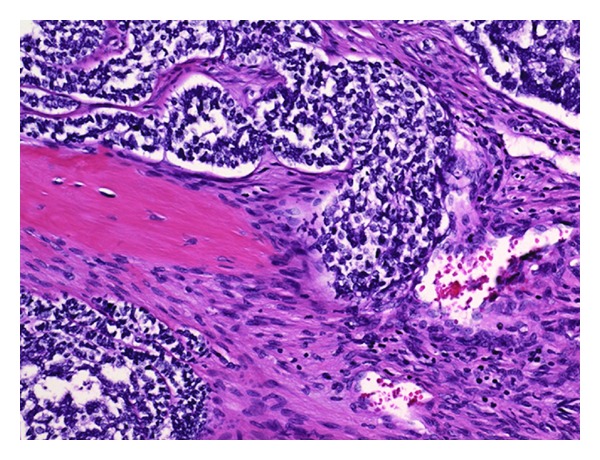
Histological preparation (200x magnification, H-E stain) which shows basaloid, epithelial cell groups intermingled with a fibrous spindle cell component.

**Figure 7:**
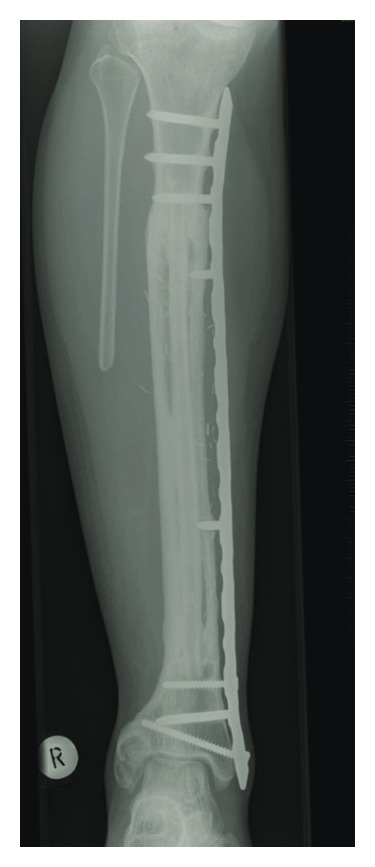
Postoperative AP radiograph at the time of 5-year followup. Surgery was performed due to multicentric adamantinoma of the right tibia and fibula. The tibial defect was bridged with a homologous tibia allograft and a vascularised fibula autograft.

Reconstruction was performed by bridging the tibia with a vascularized contralateral fibula autograft combined with a tibial massive allograft after resection of 21 cm of the tibia and 19 cm of the fibula with wide surgical margins (Figures 3 and 4). On microscopic examination, the tumour was found to consist of epithelial cells surrounded by a fibrous stroma and immunohistochemical examination found the tumour cells to be positive for cytokeratine and negative for vimentin (Figures 5 and 6). At 5 years' follow-up, the 28-year-old patient was subjectively free of complaints and there was no evidence of disease (Figure 7).

### 2.1. Tissue Processing, DNA Extraction, and PCR Analysis

Fresh frozen tissue samples, which were obtained from the three tumour locations intraoperatively and stored in a −70° freezer, were analyzed histologically and peripheral blood was also available for comparative analysis. According to a previous publication [7], we studied the X chromosome inactivation pattern focusing particularly on the methylation-sensitive polymerase chain reaction and primers that target the polymorphic CGG trinucleotide repeat of FMR1 gene and the polymorphic CAG repeat, on exon 1 of the human androgen receptor gene (AR).

**Figure 8:**
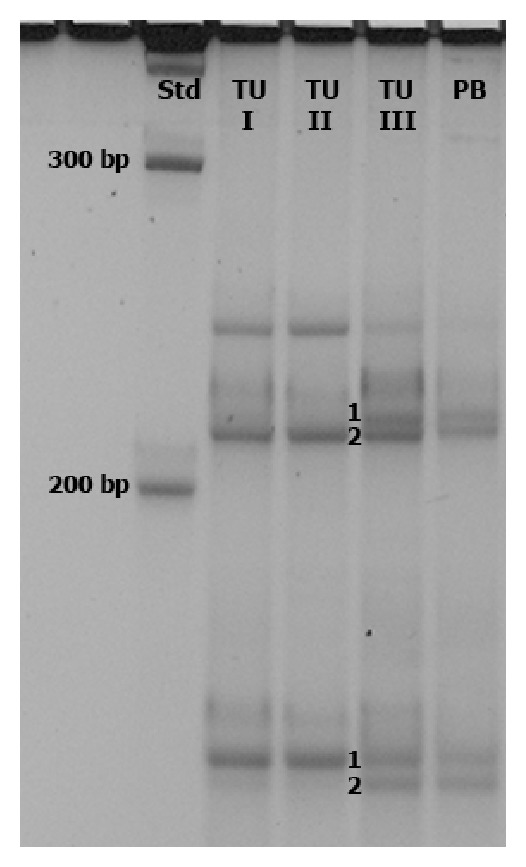
Analysis of electrophoretic band patterns: X chromosome inactivation pattern according to a methylation-sensitive PCR of the *FMR1* gene region. A 100% skewing of the tibia tumour (TU I) as well as the fibula tumour (TU II) could be detected (presence of a single cell line consisting of the methylated (M) allele 2 and the unmethylated (UM) allele 1). The analysis of the distal lesion of the tibia (TU III) as well as the peripheral blood (PB) showed a random X inactivation (four distinct bands of equal intensity that represent the two methylated and unmethylated alleles).

Analysis found the AR to be homozygous and therefore not informative. Studying the FMR1 gene, we detected a 100% skewing of the X inactivation pattern of both locations, the right distal fibular lesion and the right proximal tibial lesion, and found that the same allele was methylated (Figure 8). The samples taken from a radiologically suspected but histologically unverified skip lesion located more distally and samples from the peripheral blood showed a random X inactivation pattern.

## 3. Discussion

Adamantinoma of the long bones, which was first named and described by Fischer in 1913 [8], metastasizes in 12–29% of cases. It was named adamantinoma because it resembled adamantinoma found in the jaw that is now referred to as ameloblastoma [9]. The tumour can spread to regional lymphatic nodes and to the lungs, more infrequently to the skeleton, liver, and brain [1]. The differentiation between low-grade malignant adamantinoma and the benign osteofibrous dysplasia (OFD), which was first described by Campanacci in 1976 [10], can be difficult. Both classic and osteofibrous-like adamantinomas show recurring numerical chromosomal abnormalities. A review of literature revealed that extra copies of chromosomes 7, 8, 12, 19, and 21 are recurrent in adamantinoma [11–13]. Extra copies of the same chromosomes with the exception of chromosome 19 have been also found in OFD [11]. This fact also indicates a possible and often discussed relationship between adamantinoma and OFD.

The objective of the present study was to define the origin of the fibular lesion. Whether tibial and fibular lesions represent monoclonal or polyclonal proliferations is not yet exactly known. Neither could we find evidence in literature whether the fibular lesion results from an independent origin (de novo) or whether it is a metastatic lesion. It is only known that adamantinoma of the tibia is frequently combined with one or more lesions in the ipsilateral fibula, but, as stated before, clonality of the fibular tumour has not yet been assessed.

In each female cell one of the maternally or paternally derived X chromosomes is becoming inactive and the random inactivation occurs in the majority of females (Lyon hypothesis) [14]. The choice of chromosomes is made independently and with an equal probability factor. The “inactive” or “nonworking” X chromosome is, however, only genetically inactive, otherwise it replicates normally [14]. However, preferential selection of X chromosome inactivation only occasionally occurs, for instance in neoplasms which proliferate in a clonal manner.

In our opinion the analyses performed can be helpful in evaluating and answering our question. Clonality analyses can be a helpful tool in order to distinguish multicentricity from multifocality in benign or malignant processes, for example, in soft tissue lesions as well as in thyroid tumours [7, 15, 16]. The concept of the monoclonal nature of solid tumours has been questioned by authors such as Schwartz et al., who presented data showing that giant-cell tumour of bone is polyclonal in its matter of proliferation [15]. As these authors did not differentiate between the tumorous stromal cells and the presumably reactive multinucleate giant cells, their results are questionable. 

**Figure 9:**
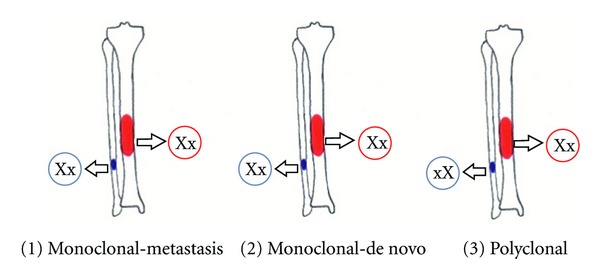
Schematic illustration of possible clonal origin of two lesions in case of multifocal/multicentric adamantinoma. The Xx and xX are representing the random X inactivation pattern in the respective locations.

Unfortunately, in the present case our findings are no valid contribution to help to explain the origin of the fibular lesions. Although the results of the analysis are concordant with a regional metastasis by telling us that the same allele in both expansions is methylated, this single case cannot corroborate that thesis, simply, because of the 50% chance of a de novo genesis that persists in a monoclonal result (Figure 9). Further analyses of X inactivation patterns of future polyclonal cases of adamantinoma may, thus, shed more light on this topic.
